# Mental health help-seeking among Korean men: the influence of stigma, masculine norms, and face

**DOI:** 10.1186/s40359-025-02793-y

**Published:** 2025-05-02

**Authors:** Soomin Kim, Dongil Kim

**Affiliations:** College of Education, Seoul National University, Seoul, Republic of Korea

**Keywords:** Loss of face, Stigma, Attitudes toward seeking professional psychological help, Masculine norms

## Abstract

**Background:**

Previous studies have found that Asians and men are more susceptible to the stigma and stereotypes associated with mental illness. This social perception makes it difficult for Korean men to seek professional psychological help even when they desperately need it. The primary objectives of this study were to investigate barriers to mental health help-seeking among Korean men and to identify the role of sociocultural factors.

**Methods:**

A total of 341 Korean men aged 19 to 59 years participated in an online survey, which included questionnaires on loss of face, self-stigma of seeking help, attitudes toward seeking professional psychological help, and conformity to masculine norms. Structural Equation Modeling was used to explore the relationships among these variables.

**Results:**

The results revealed that loss of face indirectly affected attitudes toward seeking professional psychological help (ATSPPH) through its mediating effect on self-stigma. Loss of face did not directly influence ATSPPH. Furthermore, conformity to masculine norms negatively affected ATSPPH, and self-stigma mediated this relationship.

**Conclusion:**

These findings highlight the importance of understanding the influence of sociocultural factors in encouraging Korean men to seek help. The ultimate goal is to foster a social environment that encourages open discussion about mental illness, masculinity, and Asian values.

## Background

In contrast to the impressive economic development that South Korea has achieved over the past several decades, the country has notably led in suicide rates among Organization for Economic Co-operation and Development (OECD) nations as of 2024, signaling a critical need for attention to mental health issues. Particularly affected are men who face significant challenges when seeking psychological support. The suicide rate for Korean men is 2.3 times higher than that for women [1]. However, a survey by the Korea National Mental Health Center in 2021 revealed that only 68% of men were willing to consult a psychologist or counselor when experiencing mental illness, compared to 85% of women [1]. This discrepancy raises the following question: What specific barriers to mental healthcare do Korean men face?

The Theory of Planned Behavior (TPB) [2] posits that an individual's intention to perform a behavior is the most immediate predictor of that behavior, and that such intentions are shaped by three key factors: attitudes toward the behavior, subjective norms, and perceived behavioral control. Attitudes reflect the individual’s evaluation of the behavior as positive or negative; subjective norms represent perceived social pressures; and perceived behavioral control refers to the perceived ease or difficulty of performing the behavior [2].

In the context of mental health help-seeking, TPB provides a robust framework for understanding how sociocultural variables, such as *loss of face* and *masculine norms*, indirectly influence intentions through their effects on attitudes and subjective norms. Individuals with strong face concerns may perceive psychological help-seeking as a threat to personal and familial reputation, leading to negative evaluations of the behavior [3, 4] Similarly, adherence to traditional masculine norms—which emphasize stoicism, emotional control, and self-reliance—may foster negative attitudes toward help-seeking by framing it as inconsistent with socially endorsed masculine ideals [5, 6].

Moreover, both loss of face and masculine norms can shape subjective norms by reinforcing cultural and gender-based expectations that discourage emotional disclosure and reliance on external support [7, 8]. In collectivist societies such as Korea, help-seeking is not merely a personal act but one embedded in relational and reputational contexts, further complicating intentions to seek support. By embedding these cultural constructs into the TPB framework, the present study seeks to clarify how culturally internalized beliefs influence Korean men’s reluctance to pursue professional psychological help.

Previous research has identified stigma, loss of face, and masculine norms as significant barriers to mental health help-seeking [9–11]. However, limited studies have explored how these factors collectively shape attitudes, particularly among Korean men. This study aims to bridge this gap by examining the influence of loss of face, self-stigma, and conformity to masculine norms on Korean men’s attitudes toward seeking professional psychological help (ATSPPH). The findings will inform culturally sensitive interventions that promote mental health support while addressing sociocultural barriers.

### Masculinity and help-seeking

Seeking psychological help is often perceived as incompatible with the societal expectations of masculinity, leading men to internalize societal judgments of weakness and inadequacy [8–10, 12]. Traditional masculine norms, which emphasize emotional restraint, self-reliance, and fulfilling protective roles, foster self-stigma by framing help-seeking as a violation of masculine identity [9, 10, 12]. Despite the evolving concept of masculinity in contemporary society, the traditional masculine ideal in most cultural contexts is defined as being independent and emotionally stable [13].

In this dominant cultural environment, men may struggle to acknowledge their emotional distress [12]. In a meta-analytic study on the relationship between masculinity and help-seeking for depression, men demonstrated poor emotional literacy and were more likely to manifest anger, substance abuse, and conflict as expressions of their depressive state [8]. Consequently, men often label themselves as ‘stressed’ rather than depressed.

Vandello and Bosson [14] hypothesized manhood as “precarious.” Men feel the need to prove their masculinity and experience greater threats to it than women do toward their femininity. By seeking professional help, men risk being seen as weak and less likely to seek psychological intervention. Seidler et al. [8] showed that men might delay seeking professional help until their symptoms become severe. Refusing help for emotional difficulties is often viewed as a symbol of strength and a way for men to prove their masculinity and regain power [14].

These traditional masculine norms, when internalized, create a significant barrier to help-seeking by amplifying self-stigma and reinforcing the perception that addressing emotional difficulties is incompatible with societal expectations of masculinity [12, 13].

### Stigma and help-seeking

Beyond dominant gender roles, stigma has emerged as one of the most prominent potential mediating factors influencing help-seeking behavior. Public mental health stigma refers to society’s negative views toward individuals with mental illness [15], while self-stigma occurs when these societal views diminish an individual’s self-worth. Research indicates that men are more likely to internalize public stigma than women, often perceiving themselves as weak and incompetent when experiencing mental illness [8, 16]. This internalization can increase self-stigmatization, especially when psychological problems are seen as threats to masculinity, potentially exacerbating mental health issues [17].

Self-stigma has a significant negative correlation with attitudes toward seeking professional psychological help (ATSPPH) [16, 18]. A comprehensive systematic review and meta-analysis found a substantial negative association between mental health-related stigma and active help-seeking behaviors [19]. Among the most frequently reported stigma-related barriers are concerns about disclosure and fear of judgment, which often deter individuals from accessing care [20]. Recognizing and addressing these dimensions of stigma is critical to understanding and mitigating the barriers that men face when seeking mental health support.

### Cultural barriers to help-seeking: Face and collectivism

In many Asian cultural contexts, the concept of “face” plays a critical role in shaping individuals’ behaviors and decisions, particularly in relation to emotional disclosure and mental health [21]. In Korea, this notion is expressed through chemyon (체면)—a culturally embedded construct that reflects one’s social self-image and moral standing in the eyes of others [22, 23]. Chemyon is not merely a personal concern; it is relational and deeply tied to Confucian ideals of harmony, hierarchy, and group-oriented behavior [11, 22, 23]. The loss of face through revealing psychological distress is often perceived not only as a personal failure but also as bringing shame to one’s family, reinforcing avoidance of professional psychological help [24, 25].

According to Choi and Lee [23], chemyon consists of two distinct dimensions: constructive chemyon, which motivates individuals to engage in socially valued behaviors to build a good reputation, and defensive chemyon, which leads individuals to avoid behaviors that might result in shame, criticism, or the loss of social standing. While constructive chemyon can sometimes encourage adaptive social engagement, it is the defensive chemyon that more directly inhibits psychological help-seeking. Individuals influenced by defensive chemyon are likely to internalize distress and avoid mental health services for fear of social exposure or reputational damage. This fear is often exacerbated in highly interdependent cultures where identity is largely defined in relational terms [22, 27].

This dynamic is further reinforced by Korea’s collectivist orientation, where interdependence, social roles, and family honor are highly emphasized (Kim et al., 2005; Shea & Yeh [21]). Emotional expression, particularly of distress, can be viewed as disrupting group harmony or burdening others. As a result, individuals may suppress emotional needs or reframe distress in socially acceptable terms to preserve equilibrium and avoid disapproval. These tendencies are compounded by culturally sanctioned stigma and the internalization of rigid identity norms [26, 27].

Empirical research in both Korean and broader Asian contexts supports the notion that face-related concerns and culturally specific values act as barriers to psychological help-seeking. For example, Mojaverian et al. [31] found that East Asian participants were significantly less likely than Western participants to seek help when it threatened their social image. Kim and Lee [24] similarly reported that fear of losing face and violating social expectations deterred Korean participants from seeking support even in the face of significant emotional distress. Research among Asian American and Asian international students also shows that values such as emotional control, family obligation, and model minority expectations are associated with lower help-seeking attitudes and intentions [26–30].

Moreover, help-seeking among individuals from Asian backgrounds is deeply influenced by shared cultural values including relational self-construal and stigma concerns [21, 26, 27]. These patterns have been observed in immigrant populations [26, 27] college students [24, 25] and international settings [31], highlighting the widespread influence of sociocultural norms across contexts.

These findings emphasize that for Korean men, psychological help-seeking is not simply a personal or medical decision but a socially consequential act embedded in cultural values and identity regulation. Interventions must consider face, honor, and collectivist norms as core barriers and potential leverage points for change. Promoting destigmatization, normalizing emotional vulnerability, and validating help-seeking as aligned with relational and moral responsibility may be more culturally acceptable and effective [22–25]

### Current study

Studies on the impact of masculine norms on mental help-seeking are incomplete without understanding the collectivistic worldview of Asian men [32]. Although previous research has demonstrated the influence of face and masculine norms on ATSPPH [7–10, 12–14, 17, 18, 21–27], no study has explored the joint effects of face and masculine norms on professional help-seeking attitudes.

This study hypothesizes that sociocultural norms, such as face and traditional masculinity, are internalized into self-stigma, which mediates their negative influence on attitudes toward seeking professional psychological help (ATSPPH). By emphasizing how these norms operate as underlying mechanisms of self-stigma, the study aims to provide a nuanced understanding of the sociocultural factors that inhibit help-seeking behaviors among Korean men.

## Methods

### Data collection

Data were collected from 341 adult Korean men aged 19–59 using an online research company. The survey was conducted from November 9 at 6:00 PM to November 10 at 6:00 PM, 2022. The survey reach and completion rates were 27.7% and 47.9%, respectively. The survey was conducted through a research agency with a panel of over 1.7 million members, allowing for rapid participant recruitment. Within 24 h, the target sample size was reached, at which point data collection was closed. While a longer distribution period was possible, the recruitment process was designed to efficiently reach the target population within a limited timeframe. Additional information about the participants is provided in Table 1. The survey was presented in a questionnaire format that could be completed online or via a mobile device.

**Table 1:** Data acquisition information

	Number of Participants
Survey Dispatch	2788
Access	772
Completed Responses	370
Not Targeted	43
Exceeded Target	286
Midterm Withdrawal	73
Excluded Data (Over, Suspected Insincerity)	29
Total Responses	341

### Measures

#### Conformity to masculine norms

The Short Form of the Conformity to Masculine Norms Inventory (CMNI) was used to measure traditional masculinity in men [33]. The 30-item scale is derived from the original 94-item version developed by Mahalik et al. [34]. Among the various versions of the shortened CMNIs, the 30-item version showed the best model fit and was used in this study. Scores on this four-point Likert scale were significantly correlated with indicators of validity from the original CMNI, such as depression and anxiety.

The 10 subscales within the scale are emotional control, winning, playboy, violence, heterosexual self-presentation, pursuit of status, primacy of work, power over women, self-reliance, and risk taking. The sample items for each subscale are as follows: Emotional Control – “I tend to share my feelings(R)”, Winning—“I will do anything to win”, Playboy—“I would feel good if I had many sexual partners”, Violence—“I think that violence is sometimes necessary”, Heterosexual self-presentation—“It would be awful if people thought I was gay”, Pursuit of status—“Having status is not important to me (R)”, Primacy of work—“Work comes first for me”, Power over women—“The women in my life should obey me”, Self-reliance—“I never ask for help”, Risk-taking—“I take risks”.

Two bilingual graduate students and a professional translator back-translated the scale, and an Educational Counseling professor provided supervision, as no official Korean version was available. Confirmatory factor analysis (CFA) conducted using Amos yielded the following fit indices: χ^2^(138) = 352.493, *p* < 0.000; CFI = 0.899; RMSEA = 0.070; SRMR = 0.058, indicating a reasonable model fit. The scale demonstrated good internal consistency, with a Cronbach's alpha of 0.76. The subscales' alpha coefficients ranged from 0.76 to 0.89. To assess convergent validity, we examined the correlation between the total score on this scale and the General Health Questionnaire-12 (GHQ-12) total score, which measures depression and anxiety [35]. This analysis revealed a significant positive correlation (r = 0.307, *p* < 0.01), consistent with prior research on the 30-item version of the Conformity to Masculine Norms Inventory (CMNI-30) [34].

#### Loss of face-K

The Loss of Face-K scale, originally developed by Zane and Yeh [36], is a single-factor scale that measures the degree to which individuals avoid situations or behaviors that may cause loss of face. The scale comprises 21 items rated on a 7-point Likert scale, with responses ranging from “strongly disagree” to “strongly agree.” Items include statements such as “I am more affected when someone criticizes me in public than when someone criticizes me in private” and “I try to act like others to be consistent with social norms.” Higher scores on the scale reflect a greater sensitivity to loss of face. The original scale had a reliability coefficient of 0.83, and the reliability coefficient of the scale in the present study was 0.84.

#### Attitudes toward seeking professional psychological help

The professional help-seeking attitude scale developed by Fischer and Turner [37], shortened by Fischer and Farina [38] and known as the Attitudes Toward Seeking Professional Psychological Help short-form (ATSPPH-SF), was used to measure attitudes toward seeking professional psychological help. The scale comprised 10 items. Nam [39] re-examined the construct validity of the Korean scale and found two subfactors: positive attitude and necessity, and negative attitude and unnecessariness. The scale includes five positively worded items reflecting positive attitudes and necessity, such as “I would obtain psychological help if I am upset for a long time” and five negatively worded items reflecting negative attitudes and unnecessariness, such as “Psychotherapy would not have value for me.” Respondents rated each item on a 4-point Likert scale. Higher scores indicated more positive attitudes toward professional help-seeking. Previous studies revealed a significant negative correlation between help-seeking attitudes and self-stigma [9, 10, 16, 17, 19, 20]. The internal consistency of the scale in this study was 0.72. Fischer and Farina [38] study on a Western sample yielded reliability coefficients ranging from 0.82 to 0.84.

#### Self-stigma of seeking help scale

Self-stigma levels were measured using the shortened version of the Self-Stigma of Seeking Help Scale (SSOSH), which was developed by Vogel et al. [40] and translated by Lee and Son [41]. The SSOSH comprises 10 items (with five reverse-scored items) rated on a 5-point Likert scale (1 = not at all true, 5 = very true). The scale includes items such as, “If I went to a therapist, I would be less satisfied with myself”. Higher SSOSH scores indicated greater self-stigma toward seeking professional help. The scale demonstrated good internal consistency, with alpha coefficients ranging from 0.89 to 0.91, and that of the translated version was 0.83. The internal consistency in this study was 0.77 During the development of the original scale, scores on the self-stigma of seeking help scale were negatively correlated with ATSPPH and positively correlated with public stigma [40].

### Data analysis

In this study, SPSS (version 29.0) was used to conduct descriptive statistics and correlation analyses for demographic variables and the mean scores of each scale. Additionally, a simple regression analysis was performed to examine whether age significantly predicts attitudes toward seeking psychological help (ATSPPH). To further investigate the influence of education level while accounting for potential age effects, an analysis of covariance (ANCOVA) was conducted. Furthermore, a multiple regression analysis was carried out to simultaneously assess the effects of age and education level on ATSPPH. These additional analyses provided a more comprehensive evaluation of the role of demographic variables.

To explore the relationships between key variables beyond demographic characteristics, structural equation modeling was employed using AMOS 29.0. Bootstrapping techniques were applied to verify the mediating effects, ensuring robust statistical inference.

Cook’s distance, a statistical method that considers residuals and leverage to determine the influence of each observation on the estimated regression model, was used as the criterion for identifying outliers [42]. A sample of 322 cases was used for the analysis after removing 19 cases in which the distance was greater than 1 from the regression model that estimated the relationship between face and ATSPPH.

## Results

### Demographic characteristics

A total of 322 Korean men participated in the study. Participants ranged in age from 19 to 59 years (M = 40.07, SD = 9.80; Median = 40), capturing a wide span of early to late adulthood. Monthly income levels varied considerably, ranging from 0 to 3,000,000 KRW, with a mean of 442,520 KRW (SD = 432,660) and a median of 350,000 KRW. In terms of educational attainment, 67.1% held a bachelor's degree, 13.4% had a graduate degree, and 19.6% had a high school education or less.

While the sample is not nationally representative in a probabilistic sense, its demographic distribution reflects a broad cross-section of Korean adult men with diverse socio-economic and educational backgrounds. In particular, the age distribution is consistent with working-age adult men in Korea, and the inclusion of individuals across a wide income spectrum—from unemployed to relatively high earners—strengthens the generalizability of findings. However, the relatively high proportion of college-educated participants suggests that the sample may slightly overrepresent more educated individuals, a common trend in online and survey-based psychological research. This should be considered when interpreting the findings in relation to the general male population in Korea. The demographic composition ratio of survey participants is demonstrated in Table 2.

**Table 2:** Demographic characteristics

Category		Number of Cases	Percentage (%)
Age	19–39	153	47.5
	39–59	169	52.5
Highest Level of Education	High School or below	63	19.6
	University	216	67.1
	Graduate School or Higher	43	13.4

### Descriptive statistics

Descriptive statistics were calculated using SPSS 29.0 for the 322 cases. The score range, mean, standard deviation, skewness, and kurtosis of the measurements used in this study are presented in Table 3. The multivariate normality assumption was satisfied, as all skewness and kurtosis values for each variable were below two and seven, respectively.

**Table 3:** Descriptive statistics

	Range	M	SD	Skewness	Kurtosis
Loss of face	1–7	4.53	0.98	− 0.73	1.45
Conformity to masculine norms	1–4	2.30	0.65	0.32	− 0.43
Self-stigma	1–5	3.04	0.88	0.07	− 0.12
Attitude toward professional help	1–4	2.41	0.65	0.10	− 0.17

Before validating the research model, correlation analysis was conducted among the variables (Table 4). The results of the correlation analysis indicated a significant correlation between the variables (*p* < 0.01).

**Table 4:** Correlation analysis

	Loss of face	Conformity to masculine norms	Self-stigma	Attitude toward professional help
Loss of face				
Conformity to masculine norms	0.18**			
Self-stigma	0.31**	0.28**		
Attitude toward professional help	− 0.30**	− 0.40**	− 0.51**	

### Regression analysis

The regression analysis (Tables 5, 6 and 7) indicated that age does not significantly predict ATSPPH (p = 0.812), even after controlling for education level. Similarly, an ANCOVA revealed that education level does not significantly influence ATSPPH (*p* = 0.134–0.236), even after adjusting for age. These results suggest that demographic factors such as age and education level do not play a significant role in shaping help-seeking attitudes in this study.

**Table 5:** Regression analysis

Model	Predictor	β (Coef.)	SE	t	p	95% CI
Simple Regression	Age	− 0.00	0.00	− 0.54	0.59	[− 0.009, 0.005]
ANCOVA	High School and below	1.01	0.67	1.50	0.13	[− 0.312, 2.334]
	College	0.92	0.67	1.38	0.17	[− 0.393, 2.239]
	Graduate	0.80	0.68	1.19	0.24	[− 0.526, 2.130]
Multiple Regression	Age	− 0.00	0.00	− 0.24	0.81	[− 0.009, 0.007]
	High School and below	1.01	0.67	1.50	0.13	[− 0.312, 2.334]
	College	0.92	.67	1.38	0.17	[− 0.393, 2.239]
	Graduate	0.80	0.68	1.19	0.24	[− 0.526, 2.130]

**Table 6:** Regression analysis

Predictor	Direct Effect on Help-Seeking	Indirect Effect via Self-Stigma	Total Effect
Face	–	0.20	0.20
Masculine Norms	–0.38	–0.17	–0.55
Self-Stigma	–0.55	–	–0.55

**Table 7:** Mediation Results Using Bootstrapping (10,000 Resamples)

Mediated Path	Standardized Indirect Effect (β)	95% CI Lower Bound	95% CI Upper Bound	Significant (CI excludes 0)
Face → Self-Stigma → Help-Seeking	0.20	0.09	0.34	Yes
Masculine Norms → Self-Stigma → Help-Seeking	–0.17	–0.30	–0.07	Yes

### Structural equation modeling

**Fig. 1:**
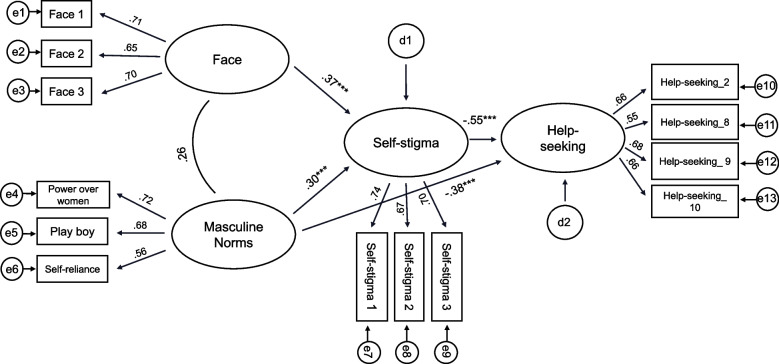
Structural equation model

To validate structural results, a measurement model was established and analyzed to examine the relationship between loss of face and ATSPPH (Fig. 1). In previous studies, three measurement variables were generated for each latent variable: loss of face scale and self-stigma scale using item parceling [43]. Additionally, based on previous research suggesting that negative attitudes toward mental health services are associated with psychological openness [44], five items reflecting negative attitudes and perceived needs were used as measurement variables for help-seeking attitudes.

Regarding the Conformity to Masculine Norms Inventory (CMNI) used in this study, although the original scale includes 10 subfactors [5] prior research has suggested that these subfactors are differentially associated with psychological outcomes. A meta-analysis of 78 samples comprising 19,453 participants found that power over women, playboy, and self-reliance were consistently and significantly associated with negative mental health outcomes, such as increased psychological distress and lower help-seeking intentions [45]. In contrast, some other subfactors, such as primacy of work, showed little to no relationship with mental health variables, suggesting that only certain masculine norms are especially relevant in this context.

Building on these findings, we selected four subfactors—power over women, playboy, self-reliance, and emotional control—to operationalize the latent variable of conformity to masculine norms. While the first three were chosen based on their consistent negative associations with psychological well-being, emotional control was included due to its widely documented role in suppressing emotional expression and discouraging help-seeking among men [9, 10, 13]. This subfactor reflects an internalized expectation to remain stoic and in control, which is particularly salient in East Asian cultural contexts, where emotional restraint is socially reinforced. Including both behavioral (e.g., power over women, playboy) and emotional (e.g., self-reliance, emotional control) aspects of masculinity enabled a more comprehensive examination of how traditional gender norms may contribute to Korean men’s reluctance to seek psychological help.

This study examined various goodness-of-fit indices commonly used in structural equation modeling for a specified model, including chi-square (${\chi }^{2}$), TLI, CFI, RMSEA, and SRMR. Using the maximum likelihood method, the initial measurement model showed inadequate fit with ${\chi }^{2}$=269.511 (*p* < 0.001), TLI = 0.782, CFI = 0.825, RMSEA = 0.083 (90% CI lower bound: 0.072, upper bound: 0.094), and SRMR = 0.084. Therefore, the fourth item of the Attitudes toward Professional Psychological Help Scale and the emotional control measurement variable of conformity to masculine norms latent variable were removed, as the standardized factor loading was below 0.5, or the explanatory power (SMC) value was below 0.4. The final measurement model demonstrated a satisfactory fit with ${\chi }^{2}$=63.661 (*p* > 0.315), TLI = 0.994, CFI = 0.995, RMSEA = 0.016 (90% CI lower bound: 0.000, upper bound: 0.039), and SRMR = 0.036. Confirmatory factor analysis revealed that all paths from the latent variables to the measurement variables were significant at the 0.001 level.

Owing to the demonstrated goodness-of-fit of the measurement model, the structural model that established causal relationships between the key variables was verified. A partial mediation model of self-stigma in the relationship between loss of face and ATSPPH was tested, with the following fit indices: ${\chi }^{2}$=63.68 (*p* > 0.315), TLI = 0.994, CFI = 0.995, RMSEA = 0.016 (90% confidence interval lower bound: 0.000, upper bound: 0.039), and SRMR = 0.036. Subsequently, a complete mediation model with the path coefficient from loss of face to ATSPPH set to 0 was verified, with the following fit indices: ${\chi }^{2}$=64.79 (*p* > 0.313), TLI = 0.994, CFI = 0.995, RMSEA = 0.016 (90% confidence interval lower bound: 0.000, upper bound: 0.038), and SRMR = 0.037, which indicated satisfactory levels of model fit. The chi-square difference test between the two models revealed that the null hypothesis was accepted (${\chi }^{2}{diff}^{(1)}$=1.11, *p* > 0.29). Given that the two models had similar goodness-of-fit indices, the competing model that provided a more parsimonious explanation for the research data was selected as the final model.

### Relationships between variables

In the research model, loss of face exerted a significant indirect effect on ATSPPH through the complete mediation of self-stigma (β = 0.37, *p* < 0.001), negatively impacting professional help-seeking directly (β = − 0.55, *p* < 0.001). This implies that higher scores for loss of face were associated with elevated levels of self-stigma of seeking help, subsequently leading to lower scores for ATSPPH. Additionally, conformity to masculine norms was found to significantly influence the self-stigma of seeking help (β = 0.30, *p* < 0.001) and ATSPPH (-0.38, *p* < 0.001).

To investigate the mediating effects of self-stigma and conformity to masculine norms on the relationship between loss of face and ATSPPH, mediation analysis was conducted using bootstrapping. A total of 10,000 samples were extracted through bootstrapping, and a 95% confidence interval was calculated using the bias-corrected method. The mediating effect of self-stigma on help-seeking in the relationship with ATSPPH for loss of face revealed a confidence interval of -0.213 to -0.065, with upper and lower bounds that did not include zero. Consequently, the mediating effect of self-stigma was statistically significant at *p* < 0.05. This confirms that self-stigma has a complete mediating effect on the relationship between loss of face and help-seeking behaviors. Furthermore, the mediating effect of self-stigma on help-seeking behaviors in the relationship with conformity to masculine norms and ATSPPH displayed a confidence interval of -0.296 to -0.068, indicating a significant mediating effect at *p* < 0.05.

## Discussion

The current study examined how face concerns, masculine norms, and self-stigma influence Korean men’s attitudes and intentions toward psychological help-seeking. The findings revealed that masculine norms exerted both direct and indirect effects on help-seeking intentions, while the effect of face concerns was mediated entirely through self-stigma. These results underscore the importance of sociocultural factors in understanding men’s reluctance to seek psychological support in the Korean context.

These relationships can be further contextualized through the Theory of Planned Behavior (TPB) [2]. In the current model, face concerns indirectly influenced help-seeking by shaping negative internalized attitudes (self-stigma), consistent with TPB’s emphasis on attitudes as a central determinant of behavioral intention. Meanwhile, masculine norms influenced intention both directly—indicating internalized normative scripts—and indirectly through their effect on self-stigma. These findings align with TPB’s framework by illustrating how cultural values and identity-related beliefs shape both attitudes and intentions toward behavior.

The full mediation effect of self-stigma between face concerns and help-seeking intention highlights how culturally embedded constructs such as Chemyon operate in the Korean male psyche. Defensive Chemyon aligns with the mechanism observed in this study, where participants with stronger face concerns reported higher self-stigma and, consequently, lower help-seeking intentions. This suggests that concerns about how one is socially perceived may lead Korean men to internalize stigma and avoid seeking help, even when needed.

Previous research has also suggested that cultural masculinity norms play a critical role in reinforcing these tendencies. Traits such as emotional control, dominance, and self-reliance are strongly emphasized within Korean masculine scripts and are often seen as incompatible with emotional openness or help-seeking. The finding that masculine norms influenced help-seeking both directly and indirectly supports prior studies showing that men who strongly endorse these norms are more likely to perceive help-seeking as a threat to their self-concept and social image [5, 10].

### Theoretical implications

These findings can be further interpreted through the lens of Social Identity [46, 47], which posits that individuals derive a sense of self from their membership in social groups and that their behaviors are influenced by the norms and expectations of those groups. This perspective is particularly relevant to the internalization of masculine norms as part of men’s social identity. Men who strongly identify with traditional masculine roles may perceive seeking psychological help as a violation of group norms, thereby jeopardizing their in-group status. Kantar and Yalçın [48] elaborate on the role of masculinity within Social Identity Theory, emphasizing how conformity to traditional masculine norms acts as a significant barrier to help-seeking. These norms create self-stigma, whereby men perceive seeking psychological help as inconsistent with their social identity, leading to avoidance behaviors. Furthermore, Social Identity Theory helps explain why some men resist psychological help even when experiencing significant distress, as they strive to maintain the social image associated with masculinity. This theoretical framework enriches our understanding of the barriers to help-seeking by situating the findings within broader sociocultural dynamics.

These insights are further deepened by Precarious Manhood Theory [14], which conceptualizes manhood as an elusive, tenuous, and socially conferred status that must be continually earned and defended. Unlike womanhood, which is often viewed as a natural and stable identity, manhood is perceived as a status that is "hard won and easily lost." According to this framework, men experience ongoing anxiety over their masculine status and are motivated to engage in behaviors that affirm masculinity while avoiding those that could be perceived as feminine or weak.

In Korean cultural contexts, this sense of precarious manhood is further intensified by collectivistic norms, strong gender-role expectations, and public visibility of one’s social standing (e.g., through Chemyon). The masculine norms endorsed in Korean society—emphasizing emotional control, self-reliance, and dominance—are not only culturally normative but also socially policed. This aligns with Precarious Manhood Theory’s assertion that men are especially sensitive to status threats and will often avoid behaviors that might signal gender-role nonconformity.

Moreover, in line with this theory, our findings showed that self-stigma acts as a psychological mechanism through which Korean men avoid help-seeking in order to maintain their social image and masculine status. For men operating under precarious manhood norms, seeking help may not simply be an issue of personal preference or lack of resources, but rather a strategic avoidance of social devaluation. Thus, Korean men’s help-seeking behavior can be better understood as a culturally specific performance of masculinity—regulated not only by internalized norms but also by external expectations of continuous masculinity validation.

Together, these theoretical interpretations illustrate that psychological help-seeking among Korean men cannot be fully understood through individual-level variables alone. Rather, it is a culturally embedded, identity-regulated behavior that reflects broader social expectations and anxieties surrounding masculinity. Future interventions should consider leveraging these theories to design programs that challenge traditional gender norms and reduce the stigma associated with help-seeking.

### Practical implications

Traditional masculine norms, such as emotional control and self-reliance, further contribute to self-stigma and reduce help-seeking behaviors in men [17]. To address these barriers, interventions focusing on self-compassion can be highly effective. Research has demonstrated that self-compassion can buffer the relationship between masculine norms and stigma, reducing perceptions of help-seeking as emasculating and fostering greater openness to psychological support [48].

A practical and evidence-based example is the “Making Peace with Yourself” program developed by Germer and Neff [49] an 8-week group-based self-compassion training that has shown effectiveness in reducing shame and increasing emotional acceptance. This curriculum can be adapted to Korean cultural contexts by emphasizing relational duty and family well-being, framing help-seeking as a way to sustain one’s capacity to fulfill social roles. A workplace adaptation of this program may be particularly impactful in male-dominated environments, where emotional disclosure is often stigmatized.

In addition, a recent study by Finlay-Jones et al. [50] tested the effectiveness of a web-based self-compassion intervention for young people with chronic health conditions. Their randomized controlled trial found significant improvements in emotional well-being and self-compassion. Given Korea’s strong digital infrastructure and high mobile literacy, such interventions could be tailored for Korean men—particularly university students and young professionals—through mobile mental health apps (e.g., Mind Cafe, WELT) or university counseling centers.

Community-based approaches that incorporate respected leaders or peer mentors can further normalize help-seeking behaviors and reduce stigma. Group counseling and peer-support programs can offer safe environments for men to explore emotional challenges in line with collectivist cultural values that emphasize social harmony and shared experience.

Awareness campaigns are another critical tool for reducing barriers to mental health services. These campaigns should aim to redefine masculinity to include emotional vulnerability and self-care as expressions of strength. Messaging that links mental health to family and workplace responsibility may resonate more deeply with Korean men, reinforcing the idea that seeking help supports—not undermines—one’s social and familial roles.

Finally, the findings from this study may contribute to the open discussion about the masculinity scripts of Korean men. Mahalik et al. [51] pointed out that counselors often encounter masculinity “scripts” such as “strong and silent,” “aggressive,” and “independent.” Helping clients engage in a cost–benefit analysis of adhering to these scripts can promote cognitive flexibility and openness to change. When clients realize the personal toll of rigid masculine norms, they may become more receptive to self-compassion practices and help-seeking.

In summary, promoting self-compassion in a culturally attuned and contextually practical manner—through digital interventions, workplace or group formats, and culturally relevant messaging—can be an effective strategy for reducing self-stigma and encouraging help-seeking among Korean men. Mental health professionals should consider how masculine norms operate in clients’ lives and use therapeutic space to foster more flexible, compassionate understandings of masculinity that align with both personal well-being and cultural values.

### Limitations

A key limitation of this study is that the cultural constructs of face and masculinity, while central to the findings, are highly context-specific and may not fully capture their nuances within Korean society or their variability across different cultural settings. For instance, in Western cultures, “face” is often associated with self-enhancement and individual morality [52], whereas in Korean culture, it is intricately tied to social achievement and collective recognition [23]. This contextual specificity underscores the need for more refined conceptualizations of these constructs in future research.

Moreover, there may be a discrepancy between men’s actual help-seeking behavior and their ATSPPH, as only the latter was measured in the current study. In this research, ATSPPH was used as the dependent variable, assuming that attitudes predict behavior based on established theories [2]. However, prior research highlights the intention-behavior gap, where intentions predict only approximately 25% of actual behavior [53]. This gap may be attributed to external barriers such as stigma, lack of accessibility, or situational constraints, as well as individual hesitations.

Despite these limitations, it can be assumed that individuals with positive intentions are more likely to engage in corresponding behaviors; abundant research supports this notion [54]. Nonetheless, future research should aim to collect longitudinal data on both attitudes and behaviors to better understand this dynamic relationship. Investigating the factors that mediate or moderate the translation of intentions into actual help-seeking behaviors—such as stigma, logistical barriers, or cultural norms—would provide valuable insights for designing interventions that effectively promote help-seeking.

We acknowledge that the relatively low survey reach (27.7%) and completion rate (47.9%) in our study may impact the generalizability of our findings. Although we utilized South Korea's largest research panel, comprising approximately 1.7 million members, the high volume of concurrent surveys conducted by the research company likely contributed to the low reach. Additionally, the sensitive nature of the topics—mental health and masculinity—subject to cultural stigmas in Korean society, may have further reduced participation and completion rates.

While this study provides valuable insights into the attitudes of Korean men toward mental health help-seeking, its generalizability to the broader population requires careful consideration. The findings predominantly reflect the experiences and attitudes of individuals familiar with digital platforms, as the recruitment process relied on online surveys and excluded individuals over the age of 59. Consequently, the study may not capture the perspectives of older adults, less educated individuals, or those with limited access to technology—groups that may exhibit different attitudes and behaviors toward mental health help-seeking.

To address these challenges and enhance generalizability, future research should implement strategies to improve survey reach and completion rates, such as refining survey design to reduce participant burden and using proactive engagement methods. Additionally, employing more inclusive sampling methods, such as in-person surveys, community outreach, and partnerships with local organizations, could help capture the perspectives of less educated men and those from underserved communities. Incorporating qualitative methods, such as interviews or focus groups, may also provide deeper insights into the unique challenges and perspectives of less educated men regarding mental health help-seeking.

By broadening the scope of research to include these underrepresented groups, the findings can be validated and extended to the broader population of Korean men. This approach will not only improve the generalizability of the results but also inform more inclusive and effective mental health policies and interventions tailored to the needs of diverse demographic groups.

## Conclusions

Korean men’s help-seeking behavior is hindered by several socio-cultural factors, including self-stigma of seeking help, loss of face, and conformity to masculine norms. Especially, loss of face was fully mediated by self-stigma and inversely correlated with help-seeking attitude. The findings of this study suggest that understanding these influences may facilitate the development of more effective psychological interventions and foster open conversations about mental health among Korean men.

## Data Availability

The dataset is not publicly available for confidentiality reasons, but it can be made available upon reasonable request.

## Abbreviations

ATSPPH: Attitudes toward seeking professional psychological help
CMNI: Conformity to Masculine Norms Inventory
KCHS: Korean Community Health Survey
LOF-K: Loss of Face-K
OECD: Organization for Economic Co-operation and Development
SSOSH: Self-Stigma of Seeking Help Scale
TPB: Theory of Planned Behavior
